# Association Between Androgen Deprivation Therapy and Mortality Among Patients With Prostate Cancer and COVID-19

**DOI:** 10.1001/jamanetworkopen.2021.34330

**Published:** 2021-11-12

**Authors:** Andrew L. Schmidt, Matthew D. Tucker, Ziad Bakouny, Chris Labaki, Chih-Yuan Hsu, Yu Shyr, Andrew J. Armstrong, Tomasz M. Beer, Ragneel R. Bijjula, Mehmet A. Bilen, Cindy F. Connell, Scott Joseph Dawsey, Bryan Faller, Xin Gao, Benjamin A. Gartrell, David Gill, Shuchi Gulati, Susan Halabi, Clara Hwang, Monika Joshi, Ali Raza Khaki, Harry Menon, Michael J. Morris, Matthew Puc, Karen B. Russell, Neil J. Shah, Nima Sharifi, Justin Shaya, Michael T. Schweizer, John Steinharter, Elizabeth M. Wulff-Burchfield, Wenxin Xu, Jay Zhu, Sanjay Mishra, Petros Grivas, Brian I. Rini, Jeremy Lyle Warner, Tian Zhang, Toni K. Choueiri, Shilpa Gupta, Rana R. McKay

**Affiliations:** 1 Lank Center for Genitourinary Oncology, Dana-Farber Cancer Institute, Boston, Massachusetts; 2 Vanderbilt University Medical Center, Nashville, Tennessee; 3 Duke Cancer Institute Center for Prostate and Urologic Cancer, Duke University, Durham, North Carolina; 4 Oregon Health and Science University Knight Cancer Institute, Portland; 5 UPMC Western Maryland, Cumberland; 6 Winship Cancer Institute of Emory University, Atlanta, Georgia; 7 Penn State Cancer Institute, Hershey, Pennsylvania; 8 Cleveland Clinic Taussig Cancer Institute, Cleveland, Ohio; 9 Missouri Baptist Medical Center, St Louis; 10 Massachusetts General Hospital, Boston; 11 Montefiore Einstein College of Medicine, Bronx, New York; 12 Intermountain Healthcare, Salt Lake City, Utah; 13 University of Cincinnati College of Medicine, Cincinnati, Ohio; 14 Henry Ford Cancer Institute, Henry Ford Hospital, Detroit, Michigan; 15 University of Washington, Seattle Cancer Care Alliance, Fred Hutchinson Cancer Research Center, Seattle; 16 Stanford University, Stanford, California; 17 Memorial Sloan Kettering Cancer Center, New York, New York; 18 Virtua Health Network, Marlton, New Jersey; 19 Tallahassee Memorial Healthcare, Tallahassee, Florida; 20 Moores Cancer Center, University of California, San Diego; 21 University of Kansas Medical Center, Westwood

## Abstract

**Importance:**

Androgen deprivation therapy (ADT) has been theorized to decrease the severity of SARS-CoV-2 infection in patients with prostate cancer owing to a potential decrease in the tissue-based expression of the SARS-CoV-2 coreceptor transmembrane protease, serine 2 (TMPRSS2).

**Objective:**

To examine whether ADT is associated with a decreased rate of 30-day mortality from SARS-CoV-2 infection among patients with prostate cancer.

**Design, Setting, and Participants:**

This cohort study analyzed patient data recorded in the COVID-19 and Cancer Consortium registry between March 17, 2020, and February 11, 2021. The consortium maintains a centralized multi-institution registry of patients with a current or past diagnosis of cancer who developed COVID-19. Data were collected and managed using REDCap software hosted at Vanderbilt University Medical Center in Nashville, Tennessee. Initially, 1228 patients aged 18 years or older with prostate cancer listed as their primary malignant neoplasm were included; 122 patients with a second malignant neoplasm, insufficient follow-up, or low-quality data were excluded. Propensity matching was performed using the nearest-neighbor method with a 1:3 ratio of treated units to control units, adjusted for age, body mass index, race and ethnicity, Eastern Cooperative Oncology Group performance status score, smoking status, comorbidities (cardiovascular, pulmonary, kidney disease, and diabetes), cancer status, baseline steroid use, COVID-19 treatment, and presence of metastatic disease.

**Exposures:**

Androgen deprivation therapy use was defined as prior bilateral orchiectomy or pharmacologic ADT administered within the prior 3 months of presentation with COVID-19.

**Main Outcomes and Measures:**

The primary outcome was the rate of all-cause 30-day mortality after COVID-19 diagnosis for patients receiving ADT compared with patients not receiving ADT after propensity matching.

**Results:**

After exclusions, 1106 patients with prostate cancer (before propensity score matching: median age, 73 years [IQR, 65-79 years]; 561 (51%) self-identified as non-Hispanic White) were included for analysis. Of these patients, 477 were included for propensity score matching (169 who received ADT and 308 who did not receive ADT). After propensity matching, there was no significant difference in the primary end point of the rate of all-cause 30-day mortality (OR, 0.77; 95% CI, 0.42-1.42).

**Conclusions and Relevance:**

Findings from this cohort study suggest that ADT use was not associated with decreased mortality from SARS-CoV-2 infection. However, large ongoing clinical trials will provide further evidence on the role of ADT or other androgen-targeted therapies in reducing COVID-19 infection severity.

## Key Points

**Question:**

Given the higher COVID-19–related mortality rate observed among men than among women, is androgen deprivation therapy associated with decreased rate of 30-day mortality from COVID-19 among patients with prostate cancer?

**Findings:**

In this cohort study of 1106 patients, no statistically significant difference was found in the rates of all cause 30-day mortality following COVID-19 infection among men with prostate cancer receiving androgen deprivation therapy (15%) vs those not receiving androgen deprivation therapy (14%).

**Meaning:**

The findings of this cohort study do not support an association between androgen deprivation therapy and 30-day mortality among patients with COVID-19 infection.

## Teaser

This cohort study using data from the COVID-19 and Cancer Consortium registry examines whether androgen deprivation therapy is associated with decreased rates of mortality within 30 days of COVID-19 diagnosis among patients with prostate cancer.

## Introduction

Since the recognition of SARS-CoV-2 in December 2019 in Wuhan, China, COVID-19 has rapidly spread worldwide, causing widespread disease and mortality.^1^ Patients with cancer or history of cancer experience a disproportionate burden of severe outcomes from COVID-19 infection; the risk factors associated with worse outcomes include advanced age, poor Eastern Cooperative Oncology Group (ECOG) performance status, and active cancer (compared with patients in remission).^2,3^

Male (vs female) sex is associated with higher rates of hospitalization and admission to intensive care units from COVID-19 infection.^4^ It has been hypothesized that the observed sex differences may be mediated through androgen regulation of cellular processes.^2^ Androgens and the androgen-regulated transmembrane protease, serine 2 (TMPRSS2) play an important role in prostate cancer cell invasion, tumor growth, and metastasis.^3,5^ The *TMPRSS2:ERG* gene fusion is the most frequent genomic alteration in prostate cancer, leading to an androgen-regulated fusion oncogene.^6,7^ The TMPRSS2 protein also plays a central role in SARS-CoV-2 pathogenicity; the viral spike glycoprotein is cleaved by TMPRSS2, activating SARS-CoV-2 for virus-cell fusion.^6^ Of substantial therapeutic interest is the potential for androgen deprivation therapy (ADT) to downregulate *TMPRSS2* transcription in pulmonary tissue and, in turn, reduce host susceptibility to or severity of SARS-CoV-2 infection.^4,8^ Other types of therapy, such as the use of androgen receptor inhibitors (ARIs), may also exert an effect through mechanisms associated with the androgen axis or pathway.^9^

Thus far, clinical evidence has been discordant regarding a protective role of ADT for patients with prostate cancer who develop COVID-19. Montopoli et al^10^ reported that the incidence of COVID-19 was markedly higher among men with prostate cancer not receiving ADT than among patients receiving ADT (odds ratio [OR], 4.05; 95% CI, 1.55-10.59; N = 118). In a single institution series in New York City (N = 58), Patel et al^11^ reported lower rates of hospitalization and supplemental oxygen requirements for patients receiving ADT compared with patients not receiving ADT. By contrast, Klein et al^12^ found no difference in the risk of infection for patients receiving ADT compared with those not receiving ADT (OR, 0.93; 95% CI, 0.54-1.61; *P* = .80; N = 1779). Aside from patients with prostate cancer, lower baseline testosterone values are associated with more severe COVID-19 disease in men, independent of other known risk factors associated with COVID-19 severity, suggesting a contrary hypothesis that testosterone may be protective in men.^13^

Other systemic therapies may be important in modulating the pathogenesis of SARS-CoV-2. Grivas et al^14^ reported an association between recent cytotoxic chemotherapy and adverse outcomes, but no such signal of detrimental outcomes for patients receiving endocrine therapies or immunotherapy.^4,14,15,16,17,18^ Because patients with metastatic prostate cancer may receive chemotherapy or hormonal therapies with agents targeting androgen receptors during their treatment, the interaction of these treatments may have a variable association with COVID-19 severity.

Given the possibility that ADT may be associated with the modulation of outcomes from COVID-19 infection, we performed an analysis using data from the COVID-19 and Cancer Consortium (CCC19) registry to test the primary hypothesis that ADT may have an independent association with death within 30 days after COVID-19 diagnosis for patients with prostate cancer, after adjusting for a number of additional baseline confounding factors.^19,20^

## Methods

### Study Design

This cohort study used data from the CCC19, which maintains a centralized multi-institutional registry of patients who have COVID-19 and a current or past diagnosis of cancer. The registry schema and data format have been previously described.^4,17^ The registry was built and is maintained as an electronic database using REDCap software at Vanderbilt University Medical Center in Nashville, Tennessee.^21,22^ Reports for the present study were accrued from March 17, 2020, to February 11, 2021, and included patients receiving a diagnosis of SARS-CoV-2 infection that was confirmed by polymerase chain reaction or serology tests. For propensity matching, patients without prostate cancer and those with 2 or more malignant neoplasms (synchronous or metachronous) were excluded. Reports with low-quality data (quality score >4 using our previously defined metric^23^) or incomplete outcome ascertainment resulting in unknown status of the primary outcome were also excluded. This study followed the Strengthening the Reporting of Observational Studies in Epidemiology (STROBE) reporting guideline^24^ and was approved by local institutional review boards at participating sites per institutional policy. The study was exempted by the institutional review board review of Vanderbilt University Medical Center from the requirement for obtaining informed consent because no identifiable patient information was collected. This ongoing study is registered at ClinicalTrials.gov^25^ (NCT04354701).

### Outcome Definitions

The primary outcome was death from any cause within 30 days of COVID-19 diagnosis among patients with prostate cancer receiving ADT. The comparator was patients not reported to be receiving ADT at the time of COVID-19 infection. Models were adjusted for additional baseline factors. The secondary end point was a 5-level ordinal scale of COVID-19 severity among patients receiving ADT that was based on a patient’s most severe reported disease status—defined as not admitted to the hospital (uncomplicated), admitted to the hospital, admitted to an intensive care unit, mechanically ventilated at any time after COVID-19 diagnosis, or died of any cause within 30 days of COVID-19 diagnosis. The comparator was patients not reported to be receiving ADT at the time of COVID-19 infection. Models were adjusted for additional baseline factors. We also performed a subgroup analysis to determine the comparative mortality rate within 30 days of COVID-19 diagnosis for patients receiving additional prostate cancer therapies compared with ADT alone. For the subgroup analysis, patients were grouped by first-generation ARI (ARI-1: nilutamide, bicalutamide, and flutamide), second-generation ARI (ARI-2: darolutamide, enzalutamide, and apalutamide), abiraterone acetate in combination with prednisone, and cytotoxic chemotherapy. Patient receipt of systemic therapies was defined as administration within 3 months prior to presentation with COVID-19 infection. Receipt of ADT was defined as prior bilateral orchiectomy or as a gonadotropin-releasing hormone analogue or antagonist administered within 3 months of COVID-19 diagnosis given that the vast majority of administered ADT is long acting.^26^

### Statistical Analysis

All statistical methods were specified before database lock (February 11, 2021) and the subsequent initiation of the analysis. Standard descriptive statistics were used to summarize the baseline demographic characteristics of the cohort.

Before conducting multivariable data analyses to evaluate the primary hypothesis, we performed multiple imputation (with 10 imputations) for the missing values using additive regression, bootstrapping, and predictive mean matching. For the primary end point, to reduce the overall imbalance of the confounding variables among the study groups in this nonrandomized study (eTable 1 in the Supplement), we used propensity score matching (PSM) to balance the covariate distributions in the 2 ADT groups. The unmatched data were not used in subsequent regression analyses.

The propensity matching used the nearest-neighbor method with a 1:3 ratio of treated units to control units and without replacement (control units were matched to only 1 treated unit each). For the 1:3 matching, we adopted variable ratio matching, that is, up to 3 control units were matched to each treated unit, an approach that has been shown to have better bias reduction properties.^27^ The balanced covariates were age, body mass index, race and ethnicity (self-identified as Hispanic, non-Hispanic Black, and non-Hispanic White), ECOG performance status (≥2 vs 0 or 1), smoking status (current or former vs never), comorbidities (presence vs absence for each of heart disease, lung disease, kidney disease, or diabetes), cancer status (in remission or stable vs active or progressing), baseline steroid use (prednisolone equivalent >10 mg daily), COVID-19 treatment (remdesivir, hydroxychloroquine, or azithromycin), and presence of metastatic disease.

Variable selection was performed using elastic-net regularization (with a mixing parameter of 1 least absolute shrinkage and selection operator) for multivariable logistic regression models (eMethods; eFigures 2 and 3 in the Supplement). However, the variable selection method selected different variables on different multiply imputed data sets. To determine a set of common variables for subsequent multivariable logistic regression models, we first applied the variable selection method to the 10 imputed data sets and then selected the variables that were picked more than 9 times. Analyses (PSM plus variable selection plus multivariable logistic regression analysis) were conducted for each of the 10 imputed data sets. The analyses for the secondary end point followed the same procedures as the primary end point.

In subgroup analysis, we focused on the cohort receiving ADT and compared the rates of 30-day mortality for patients receiving additional prostate cancer therapies, grouped by androgen receptor–targeted agent, abiraterone in combination with prednisone, and chemotherapy, compared with ADT alone. The analyses for the rates of 30-day mortality and the severity of COVID-19 disease in the 3 pairs of treatment comparisons followed the same procedure: missing imputation plus PSM (or without PSM) between the treatment groups of each pair comparison plus variable selection plus logistic regression analysis. All data analyses were performed using base R, version 3.6.1, and the R packages Hmisc, version 4.4.2, MatchIt, version 3.0.2, ordinalNet version 2.9, and glment, version 3.0-2 (R Project for Statistical Computing).

## Results

We identified 1228 men with a diagnosis of prostate cancer, of whom 1106 were included in our analysis after exclusions (eFigure 1 in the Supplement). Before PSM, the median age was 73 years (IQR, 65-79 years), and 104 patients (9%) were Hispanic, 258 (23%) non-Hispanic Black, and 561 (51%) non-Hispanic White race and ethnicity (eTable 1 in the Supplement). Overall, 266 patients (24%) had received ADT within 3 months of COVID-19 diagnosis (including 5 patients with prior bilateral orchiectomy), and 143 patients (13%) received additional prostate cancer therapies within 3 months of COVID-19 diagnosis; 158 patients (14%) died of any cause within 30 days. Additional baseline characteristics are summarized in eTable 1 in the Supplement. Before PSM, the groups were balanced between the those receiving ADT and those not receiving ADT, with the exception of a higher proportion of patients in the group receiving ADT with active cancer (216 of 266 [81%] vs 212 of 840 [25%]) and with higher rates of metastatic disease (149 of 266 [56%] vs 65 of 840 [8%]).

Before PSM, the rates of 30-day mortality were 13% (112 of 840) for patients not receiving ADT vs 17% (46 of 266) for patients receiving ADT (χ^2^ = 2.59; *df* = 1; *P* = .11). After PSM, the rates of 30-day mortality were 14% (44 of 308) for patients not receiving ADT vs 15% (25 of 169) for patients receiving ADT (χ^2^ = 0.02; *df* = 1; *P* = .88) (Table 1). The adjusted OR (aOR) for receiving ADT compared with not receiving ADT was 0.77 (95% CI, 0.42-1.42) (Table 2), also indicating that there was no significant difference for the primary end point of death from any cause within 30 days based on receipt of ADT.

**Table 1.** Descriptive Statistics of the Matched Data Based on 1 of 10 Imputed Data Sets

Characteristic	No. (%) of patients	
	Not receiving ADT (n = 308)	Receiving ADT (n = 169)
All-cause mortality at 30 d	44 (14)	25 (15)
COVID-19 severity ordinal scale		
0 (Uncomplicated)	136 (44)	66 (39)
1 (Hospitalized)	96 (31)	55 (33)
2 (Intensive care unit)	8 (3)	5 (3)
3 (Mechanical ventilation)	13 (4)	8 (5)
4 (Death within 30 d)	44 (14)	25 (15)
Unknown or missing	11 (4)	10 (6)
Age, median (IQR), y	71 (64-78)	74 (65-80)
BMI (IQR)	27.9 (25.0-31.6)	27.9 (24.7-31.7)
Race and ethnicity		
Hispanic	33 (11)	21 (12)
Non-Hispanic Black	76 (25)	36 (21)
Non-Hispanic White	169 (55)	93 (55)
Other^a^	30 (10)	19 (11)
ECOG performance status score		
0	102 (33)	53 (31)
1	50 (16)	40 (24)
≥2	39 (13)	27 (16)
Unknown	117 (38)	49 (29)
Smoking status		
Current or former	151 (49)	86 (51)
Comorbidity		
Cardiovascular	114 (37)	70 (41)
Pulmonary	46 (15)	21 (12)
Kidney	54 (18)	24 (14)
Diabetes	80 (26)	40 (24)
Cancer status		
Remission or NED	60 (19)	21 (12)
Active		
Progressing	47 (15)	30 (18)
Responding	34 (11)	33 (20)
Stable	109 (35)	60 (36)
Unknown	58 (19)	25 (15)
Metastatic disease, yes	85 (28)	84 (50)
Baseline corticosteroid use (>10 mg of oral prednisolone/d), yes	61 (20)	35 (21)
COVID-19 treatment administered		
Remdesivir	29 (9)	14 (8)
Hydroxychloroquine	44 (14)	26 (15)
Azithromycin	53 (17)	29 (17)

Abbreviations: ADT, androgen deprivation therapy; BMI, body mass index (calculated as weight in kilograms divided by height in meters squared); ECOG, Eastern Cooperative Oncology Group; NED; no evaluable disease.

^a^ Other includes American Indian or Alaska Native, Asian, Native Hawaiian, or Other Pacific Islander.

**Table 2.** Results of Regression Analysis for 30-Day Mortality and COVID-19 Severity

Characteristic	Multivariable aOR (95% CI)	
	Primary outcome: 30-d mortality (binary)	Secondary outcome: COVID-19 severity (ordinal)
Received ADT		
No	1 [Reference]	1 [Reference]
Yes	0.77 (0.42-1.42)	0.98 (0.61-1.56)
Age (per 10-y increase)	1.78 (1.30-2.46)	1.59 (1.25-2.03)
Race and ethnicity		
Hispanic	Not selected^a^	Not selected^a^
Non-Hispanic Black	1.83 (0.95-3.53)	2.14 (1.27-3.62)
Non-Hispanic White	1 [Reference]	1 [Reference]
Other^b^	Not selected^a^	Not selected^a^
ECOG performance status		
0	1 [Reference]	1 [Reference]
1	Not selected^a^	Not selected^a^
≥2	5.34 (2.49-11.49)	7.16 (3.15-16.27)
Unknown	Not selected^a^	Not selected^a^
Cardiovascular comorbidity		
No	NA	1 [Reference]
Yes	Not selected^a^	1.46 (0.94-2.25)
Diabetes		
No	NA	1 [Reference]
Yes	Not selected^a^	1.71 (1.03-2.85)
Baseline corticosteroid use >10 mg of oral prednisolone/d		
No	NA	1 [Reference]
Yes	Not selected^a^	1.25 (0.68-2.29)
Metastatic disease		
No	1 [Reference]	1 [Reference]
Yes	2.52 (1.29-4.90)	1.52 (0.95-2.43)
Administered for treatment of COVID-19		
Hydroxychloroquine		
No	1 [Reference]	1 [Reference]
Yes	4.33 (2.07-9.04)	7.13 (3.59-14.17)
Azithromycin		
No	NA	1 [Reference]
Yes	Not selected^a^	1.63 (0.84-3.20)
Remdesivir		
No	NA	1 [Reference]
Yes	Not selected^a^	6.09 (2.71-13.68)

Abbreviations: ADT, androgen deprivation therapy; aOR, adjusted odds ratio; ECOG, Eastern Cooperative Oncology Group; NA, not applicable.

^a^ The variable was not selected by elastic net regularization, for example, Hispanic vs non-Hispanic White, which implies that both Hispanic and non-Hispanic White may be considered a group.

^b^ Other includes American Indian or Alaska Native, Asian, Native Hawaiian, or Other Pacific Islander.

### Sensitivity Analysis

Besides considering SD of 0.15 for PSM, we used SD of 0.2. This resulted in a larger standardized mean difference of propensity scores between the 2 ADT groups but an increase in events. With SD of 0.2, we replicated the same procedure as for the previous analysis. The results are reported in eTable 2 in the Supplement.

The regression results revealed age (per 10 years: aOR, 1.78; 95% CI, 1.30-2.46), ECOG performance status score 2 or higher (compared with ECOG score 0: aOR, 5.34; 95% CI, 2.49-11.49), receipt of hydroxychloroquine for treatment of COVID-19 (aOR, 4.33; 95% CI, 2.07-9.04), and presence of metastatic disease (aOR, 2.52; 95% CI, 1.29-4.90) as factors associated with increased rates of 30-day mortality from COVID-19 infection (Table 2).

The secondary end point was a 5-level ordinal scale of COVID-19 severity based on a patient’s most severe reported disease status among patients receiving ADT compared with those not receiving ADT at the time of COVID-19 infection. The analysis procedure was the same as the aforementioned, and the results are reported in Table 2. There was no significant difference when COVID-19 severity was compared between the patients receiving ADT and the patients not receiving ADT (aOR, 0.98; 95% CI, 0.61-1.56).

For the subgroup analysis of 30-day mortality based on receipt of additional prostate cancer therapy (patients were grouped by receipt of ARI-1 or ARI-2, abiraterone in combination with prednisone, and chemotherapy), a descriptive analysis prior to PSM is presented in Table 3 and in eTable 3 in the Supplement. Patients receiving chemotherapy within the prior 3 months had the numerically highest reported mortality rate at 28% (7 of 25) compared with 16% (28 of 174) for patients receiving ADT, 17% (7 of 42) for patients receiving abiraterone acetate, and 16% (13 of 79) patients receiving an ARI. When each prostate cancer–specific therapy was analyzed against the reference group of patients receiving ADT (with or without other prostate cancer therapies) via logistic regression analyses with variable selection, with PSM (Table 4) or without PSM (eTables 4, 5, and 6 in the Supplement), no significant difference in mortality rate was seen for any additional prostate cancer therapy.

**Table 3.** COVID-19 Severity by Receipt of Additional Prostate Cancer Therapies

COVID-19 severity ordinal scale	No. (%) of patients		
	ADT alone	ADT with additional therapy	ADT missing data on additional therapy
ARI-1 or ARI-2 (N = 266)			
No.	120	79	67
0 (Uncomplicated)	46 (38)	27 (34)	11 (16)
1 (Hospitalized)	37 (31)	27 (34)	27 (40)
2 (ICU)	3 (2)	5 (6)	21 (31)
3 (Mechanical ventilation)	7 (6)	5 (6)	0 (0)
4 (Death within 30 d)	22 (18)	13 (16)	11 (16)
Unknown or missing	5 (4)	2 (2)	5 (7)
Abiraterone in combination with prednisone (N = 266)			
No.	157	42	67
0 (Uncomplicated)	60 (38)	13 (31)	27 (40)
1 (Hospitalized)	48 (31)	16 (38)	21 (31)
2 (ICU)	7 (4)	1 (2)	0 (0)
3 (Mechanical ventilation)	9 (6)	3 (7)	3 (4)
4 (Death within 30 d)	28 (18)	7 (17)	1 (16)
Unknown or missing	5 (4)	2 (5)	5 (7)
Chemotherapy (N = 266)			
No.	174	25	67
0 (Uncomplicated)	65 (37)	8 (32)	27 (40)
1 (Hospitalized)	58 (33)	6 (24)	21 (31)
2 (ICU)	7 (4)	1 (4)	0 (0)
3 (Mechanical ventilation	9 (5)	3 (12)	3 (4)
4 (Death within 30 d)	28 (16)	7 (28)	11 (16)
Unknown or missing	7 (4)	0 (0)	5 (7)

Abbreviations: ADT, androgen deprivation therapy; ARI, androgen receptor inhibitor; ICU, intensive care unit.

**Table 4.** Results of Regression Analyses After Propensity Score Matching for 30-Day Mortality Rates Between Additional Prostate Cancer Therapies Compared With ADT

Characteristics	Multivariable aOR (95% CI)		
	ARI	Abiraterone	Chemotherapy
Received cancer therapy			
No	1 [Reference]	1 [Reference]	1 [Reference]
Yes	0.64 (0.26-1.58)	0.89 (0.21-3.82)	3.37 (0.73-15.55)
Age (per 10-y increase)	2.68 (1.56-4.60)	4.61 (1.72-12.38)	1.49 (0.59-3.79)
ECOG performance status^a^			
0, 1, and Unknown	1 [Reference]	ND	1 [Reference]
≥2	4.63 (1.83-11.75)	ND	7.53 (1.38-41.21)
COVID-19 treatment, azithromycin			
No	ND	1 [Reference]	ND
Yes	ND	4.91 (1.12-21.58)	ND

Abbreviations: ADT, androgen deprivation therapy; aOR, adjusted odds ratio; ARI, androgen receptor inhibitor; ECOG, Eastern Cooperative Oncology Group; ND, not determined.

^a^ In ECOG Performance Status, 1 vs 0 and unknown vs 0 were not selected by elastic net regularization; thus, 0, 1, and unknown were considered as a group.

## Discussion

Given the substantial risk of COVID-19 for patients with cancer, it is essential to understand the interaction between therapies and adverse outcomes to help inform clinical decision-making. The CCC19 data set is an extensive resource detailing COVID-19 outcomes for oncology patients, with granular detail on disease- and treatment-specific variables important to the daily care of patients.^17^

In the present study, we used this data set, including more than 1200 patients with prostate cancer, to examine whether ADT use was associated with a lower rate of 30-day mortality from any cause and found no significant association. Although this finding does not support the hypothesis that ADT may be useful to modulate the clinical course of SARS-CoV-2 infection, further evaluation of these interventions in a controlled clinical trial setting may explain the discordance among study results. Our findings are consistent with Klein et al,^12^ who found no significant difference, but are in contrast with study results from cohorts in Italy by Montopoli et al^10^ and in New York City by Patel et al,^11^ which both reported more favorable outcomes in the setting of ADT exposure.

The role of androgens in modulating host susceptibility and severity of infection from SARS-CoV-2 has generated intense research interest given the difference in outcomes between male and female patients after SARS-CoV-2 infection and the potential therapeutic significance if intervention with androgen directed therapies can alter COVID-19 outcomes. There are, however, numerous factors that may explain a sex bias in outcomes. Differences between female and male innate and adaptive immune systems,^28^ not all of which are subject to androgen regulation, may be involved. For example, estrogen levels, which are higher in women, may play a protective role in the immune system. Varying social practices and sex- and gender-based differences in comorbidities may also be responsible for some of the observed difference. Although androgen-mediated immune regulation is proposed as a potential explanation for sex-discordant outcomes, modulation through ADT or androgen-targeted therapies may be ineffective on clinical end points or processes responsible for gender differences independent of the proposed androgen signaling hypothesis. Notably, the previous observation that ARIs (such as enzalutamide) may inhibit the expression of TMPRSS2 in prostate cancer cells (the originating preclinical findings supporting the exploration of ADT and ARI in COVID-19) may not be relevant in pulmonary tissue, which is an anatomic site very relevant to the development of complications from SARS-CoV-2.^29^ Baratchian et al^30^ also found no evidence for increased TMPRSS2 expression in the lungs of male vs female patients or mice and an inability for treatment with enzalutamide to decrease pulmonary TMPRSS2 levels. Furthermore, there is no difference in pulmonary TMPRSS2 expression in immunohistochemical studies comparing men and women.^30^

There are characteristic differences between patients who received ADT, its use being limited to patients with active cancer (in the setting of intermediate, high-risk localized; biochemically recurrent; or metastatic disease), and patients who did not receive therapy but who had a history of prostate cancer and have been cured, are in remission, or have recurrent disease suitable for observation. Potential confounding may come from additional systemic therapies, such as chemotherapy (accounting for 25 of 266 patients in the present study cohort receiving ADT), which may cause immunosuppression and may lead to a less robust immune response against the virus. Data from the entire CCC19 cohort have been used to interrogate this potential confounder. Wise-Draper et al^31^ reported an increased rate of 30-day mortality among inpatients who had received chemotherapy less than 2 weeks prior to a COVID-19 diagnosis. In a larger analysis, Grivas et al^14^ reported an association between chemotherapy administered within 3 months of COVID-19 presentation and increased rate of 30-day mortality (aOR, 1.61; 95% CI, 1.15-2.24). Our analysis regarding chemotherapy specific to patients with prostate cancer included insufficient numbers to independently test this hypothesis without incurring wide 95% CIs.

### Limitations and Strengths

The limitations of this study include lack of testosterone levels to measure the effectiveness of ADT, retrospective design, lack of randomization and stratification, dependency on clinically annotated data (which means that potentially important variables may have not been collected), and missing and unknown data that may have associations with the results despite the robust attempt to account for them. Patients may have received ADT outside the strict treatment definition (>3 months prior to COVID-19 presentation with a castration level of testosterone) or may have been treated with intermittent ADT and still have been counted in the cohort not receiving ADT, although this scenario likely represents a small number of patients. A number of relevant selection and confounding factors, which cannot be completely matched for, may explain the differences between the patients receiving ADT and those not receiving ADT, such as the symptomatic burden of metastatic disease or the presence of active prostate cancer, which are clinical indications for patients to receive ADT, especially given that the cause of death could not always be fully ascertained. The evolving capacity and bandwidth of health care systems, the virulence of SARS-CoV-2, and other potential confounders were difficult to account for in our study. Most patients received additional prostate cancer therapies, but the majority of those treatments were directed against the androgen axis and would be expected to act in a similar fashion to ADT. Given the wide 95% CI (0.42-1.42) for the rate of all-cause 30-day mortality in the present study, a smaller effect size may be apparent that we did not have the statistical power to identify based on our sample size and the number of events. The strengths of this study included the granular details regarding prostate cancer–specific and COVID-19–specific variables, rigorous data quality control, and large patient numbers across numerous sites.

## Conclusions

After PSM, no significant difference in the all-cause 30-day mortality rate following COVID-19 infection or in COVID-19 severity was associated with the receipt of ADT. These findings do not support the hypothesis that ADT may be useful in reducing the mortality or severity of SARS-CoV-2 infection. We await the results of ongoing prospective studies exploring the role of ADT in modulating the course and outcomes of SARS-CoV-2 infection.

## Notes

Supplement.
eMethods.
**eTable 1.** Descriptive Statistics by ADT Exposure State Before Matching **eTable 2.** Results of Regression Analysis for 30-Day Mortality and COVID-19 Severity **eTable 3.** Descriptive Statistics in the ADT Cohort **eTable 4.** Results of Regression Analysis for 30-Day Mortality Between ADT + ARI Compared to ADT, Adjusting for the Variables Selected by the Elastic-Net Regularization With a Mixing Parameter of 1 (LASSO) **eTable 5.** Results of Regression Analysis for 30-Day Mortality Between ADT + Abiraterone Compared to ADT, Adjusting for the Variables Selected by the Elastic-Net Regularization With a Mixing Parameter of 1 (LASSO) **eTable 6.** Results of Regression Analysis for 30-Day Mortality Between ADT + Chemotherapy Compared to ADT, Adjusting for the Variables Selected by the Elastic-Net Regularization With a Mixing Parameter of 1 (LASSO) **eFigure 1.** Patient Selection **eFigure 2.** Loss of Dead30 Events (All Cause 30-Day Mortality) and Standardized Mean Difference of Propensity Scores Between the 2 ADT Groups (on ADT and Not on ADT) **eFigure 3.** Distributions of Propensity Scores of Patients on ADT and Not on ADT Before and After Matching
