# Omitted Group Name, Author, Nonauthor Collaborators, Disclosure, and Grant/Support Information

**DOI:** 10.1001/jamanetworkopen.2021.45075

**Published:** 2021-12-28

In the Original Investigation titled “Association Between Androgen Deprivation Therapy and Mortality Among Patients With Prostate Cancer and COVID-19,”^1^ published November 12, 2021, the following information was omitted by the authors before publication: “for the COVID-19 and Cancer Consortium” from the end of the byline; Dimpy P. Shah, MD, PhD, from the list of authors; author affiliation; academic degrees; the list of nonauthor collaborators from the Supplement; an additional disclosure for 1 of the coauthors; and additional grant and support information from the funding statement. The corresponding author has explained these omissions in a Comment posted with the article^2^ and requested that the article be corrected.
