# Supplementary material for: Association Between Androgen Deprivation Therapy and Mortality Among Patients With Prostate Cancer and COVID-19
Source: JAMA Netw Open. 2021 Nov 12;4(11):e2134330. doi: 10.1001/jamanetworkopen.2021.34330 (PMC8590166; doi:10.1001/jamanetworkopen.2021.34330)
Supplement: Supplement. — eMethods. eTable 1. Descriptive Statistics by ADT Exposure State Before Matching eTable 2. Results of Regression Analysis for 30-Day Mortality and COVID-19 Severity eTable 3. Descriptive Statistics in the ADT Cohort eTable 4. Results of Regression Analysis for 30-Day Mortality Between ADT + ARI Compared to ADT, Adjusting for the Variables Selected by the Elastic-Net Regularization With a Mixing Parameter of 1 (LASSO) eTable 5. Results of Regression Analysis for 30-Day Mortality Between ADT + Abiraterone Compared to ADT, Adjusting for the Variables Selected by the Elastic-Net Regularization With a Mixing Parameter of 1 (LASSO) eTable 6. Results of Regression Analysis for 30-Day Mortality Between ADT + Chemotherapy Compared to ADT, Adjusting for the Variables Selected by the Elastic-Net Regularization With a Mixing Parameter of 1 (LASSO) eFigure 1. Patient Selection eFigure 2. Loss of Dead30 Events (All Cause 30-Day Mortality) and Standardized Mean Difference of Propensity Scores Between the 2 ADT Groups (on ADT and Not on ADT) eFigure 3. Distributions of Propensity Scores of Patients on ADT and Not on ADT Before and After Matching [file jamanetwopen-e2134330-s001.pdf]

Supplementary Online Content

Schmidt AL, Tucker MD, Bakouny Z, et al. Association between androgen deprivation therapy and mortality among patients with prostate cancer and COVID-19. *JAMA Netw Open*. 2021;4(11):e2134330. doi:10.1001/jamanetworkopen.2021.34330

eMethods.

eTable 1. Descriptive Statistics by ADT Exposure State Before Matching

eTable 2. Results of Regression Analysis for 30-Day Mortality and COVID-19 Severity

eTable 3. Descriptive Statistics in the ADT Cohort

eTable 4. Results of Regression Analysis for 30-Day Mortality Between ADT + ARI Compared to ADT, Adjusting for the Variables Selected by the Elastic-Net Regularization With a Mixing Parameter of 1 (LASSO)

eTable 5. Results of Regression Analysis for 30-Day Mortality Between ADT + Abiraterone Compared to ADT, Adjusting for the Variables Selected by the Elastic-Net Regularization With a Mixing Parameter of 1 (LASSO)

eTable 6. Results of Regression Analysis for 30-Day Mortality Between ADT + Chemotherapy Compared to ADT, Adjusting for the Variables Selected by the Elastic-Net Regularization With a Mixing Parameter of 1 (LASSO)

eFigure 1. Patient Selection

eFigure 2. Loss of Dead30 Events (All Cause 30-Day Mortality) and Standardized Mean Difference of Propensity Scores Between the 2 ADT Groups (on ADT and Not on ADT)

eFigure 3. Distributions of Propensity Scores of Patients on ADT and Not on ADT Before and After Matching

This supplementary material has been provided by the authors to give readers additional information about their work.

eMethods.

To determine an optimal multiplier of the standard deviation for drawing the control units in the matching, we minimized the loss of 30-day mortality events under the standardized mean difference of the propensity scores less than or close to 0.1 between the two groups. The optimal value of 0.15 was selected **(Supplementary Figure 2)**. The densities of the propensity scores between the two ADT groups after matching were clearly more similar **(Supplementary Figure 2)** and the distributions of the covariates were better balanced **(Table 1)**, compared with those before matching **(Supplementary Table 1 and Supplementary Figure 3)**. The size of the matched data was about 477 and the number of mortality events was about 69, varying with the 10 imputed datasets. Then, we performed variable selection using the elastic-net regularization (with a mixing parameter of 1, least absolute shrinkage and selection operator) for multivariable logistic regression models, due to the limited number of events in the matched data **(Table 1)**. Analyses (PSM + variable selection + multivariable logistic regression analysis) were run on each of the 10 imputed datasets. After the 10-run analyses, the average results were reported in **Table 2**.

© 2021 Schmidt AL et al. *JAMA Network Open*.

eTable 1. Descriptive Statistics by ADT Exposure State Before Matching (*N* = 1106)

	Not on ADT (*N*=840)	Receiving ADT (*N*=266)
**30-day all-cause mortality**	**112 (13)**	**46 (17)**
COVID-19 severity: Ordinal Scale		
0 - uncomplicated	336 (40)	100 (38)
1 - hospitalized	276 (33)	85 (32)
2 - ICU	30 (4)	8 (3)
3 - mechanical ventilation	46 (5)	15 (6)
4 - death within 30 days	112 (13)	46 (17)
Unknown/missing	40 (5)	12 (4)
Age median, years (IQR)	72 (65-79)	74 (65-80)
**Body Mass Index** (IQR) *[251 missing]	28.0 (25.1-31.6)	27.9 (24.9-31.4)
**Race and ethnicity**		
Hispanic	69 (8)	35 (13)
Non-Hispanic Black	203 (24)	55 (21)
Non-Hispanic White	431 (51)	130 (49)
Other*	82 (10)	23 (9)
Missing	55 (7)	23 (9)
**ECOG Performance Status**		
0	259 (31)	81 (30)
1	112 (13)	67 (25)
≥2	88 (10)	46 (17)
Unknown	338 (40)	54 (20)
Missing	43 (5)	18 (7)
**Smoking Status**		
Never	422 (50)	112 (42)
Current or Former	352 (42)	122 (46)
NA	66 (8)	32 (12)
**Cardiovascular comorbidity**		
No	492 (59)	140 (53)
Yes	303 (36)	108 (41)
Missing	45 (5)	18 (7)
**Pulmonary comorbidity**		
No	673 (80)	212 (80)
Yes	122 (15)	36 (14)
Missing	45 (5)	18 (7)
**Renal comorbidity**		
No	633 (75)	203 (76)
Yes	162 (19)	45 (17)
Missing	45 (5)	18 (7)
**Diabetes**		
No	534 (64)	187 (70)
Yes	261 (31)	61 (23)
Missing	45 (5)	18 (7)
**Cancer Status**		

© 2021 Schmidt AL et al. *JAMA Network Open*.

Remission/NED	546 (65)	21 (8)
Active, progressing	49 (6)	57 (21)
Active, responding	34 (4)	71 (27)
Active, stable	129 (15)	88 (33)
Unknown	80 (10)	25 (9)
NA	2 (0)	4 (2)
**Cancer Status - Metastatic disease present**		
No	692 (82)	80 (30)
Yes	65 (8)	149 (56)
Missing	83 (10)	37 (14)
**Baseline corticosteroid use (>10mg oral prednisolone / day)**		
No	616 (73)	183 (69)
Yes	135 (16)	54 (20)
Missing	89 (11)	29 (11)
**COVID-19 treatment administered**		
**Remdesivir**		
No	679 (81)	217 (82)
Yes	73 (9)	20 (8)
NA	88 (10)	29 (11)
**Hydroxychloroquine**		
No	614 (73)	198 (74)
Yes	137 (16)	39 (15)
Missing	89 (11)	29 (11)
**Azithromycin**		
No	600 (71)	196 (74)
Yes	151 (18)	41 (15)
Missing	89 (11)	29 (11)
**Additional prostate cancer therapies**		
**1st Generation Androgen Receptor Inhibitor (ARI-1)**		
No	135 (16)	155 (58)
Yes	3 (0)	44 (17)
Missing	702 (84)	67 (25)
**2nd Generation Androgen Receptor Inhibitor (ARI-2)**		
No	136 (16)	162 (61)
Yes	2 (0)	37 (14)
Missing	707 (84)	67 (25)
**Abiraterone**		
No	137 (16)	157 (59)
Yes	2 (0)	42 (16)
Missing	702 (84)	67 (25)
**Chemotherapy**		
No	134 (16)	174 (65)
Yes	4 (0)	25 (9)
Missing	702 (84)	67 (25)

© 2021 Schmidt AL et al. *JAMA Network Open*.

ICU: intensive care unit, NA: not available. NED: no evaluable disease, ARI; androgen receptor inhibitor, ARI-1; 1st Generation Androgen receptor inhibitor; bicalutamide, nilutamide, flutamide, ARI-2; 2nd Generation Androgen receptor inhibitor; apalutamide, darolutamide, enzalutamide, abiraterone.

*Other includes American Indian/Alaska Native, Asian, Native Hawaiian or Other Pacific Islander

© 2021 Schmidt AL et al. *JAMA Network Open*.

eTable 2. Results of Regression Analysis for 30-Day Mortality and COVID-19 Severity [propensity score matching with the caliper = 0.20].

**Characteristics**	**Multivariable AOR (95% CI) Primary outcome: 30-day mortality (binary)**	**Multivariable AOR (95% CI) Secondary outcome: COVID-19 severity (ordinal)**
**On ADT (ref = Not on ADT)**		
Yes	0.78 (0.41-1.47)	0.97 (0.61-1.53)
**Age (per 10 years increase)**	**1.78 (1.29-2.47)**	**1.58 (1.22-2.05)**
**Race (reference = Non-Hispanic White)**		
Hispanic	-	-
Non-Hispanic Black	1.86 (0.98-3.54)	**2.00 (1.18-3.39)**
Other*	-	-
**ECOG Performance Status (ref = 0)**		
1	-	-
≥2	**5.41 (2.61-11.20)**	**7.19 (3.68-14.05)**
Unknown	-	-
**Cardiovascular comorbidity (ref = No)**		
Yes	-	1.49 (0.95-2.31)
**Diabetes (ref = No)**		
Yes	-	**1.71 (1.00-2.92)**
**Baseline corticosteroid use >10mg oral prednisolone / day (ref = No)**		
Yes	-	-
**Metastatic Disease (ref = No)**		
Yes	**2.52 (1.37-4.65)**	**-**
**Therapies administered for COVID-19**		
**Hydroxychloroquine (ref = No)**		
Yes	**4.80 (2.37-9.70)**	**9.71 (5.38-17.53)**
**Azithromycin (ref = No)**		
Yes	-	-
**Remdesivir (ref = No)**		
Yes	**-**	**7.33 (3.78-14.24)**

AOR; Adjusted odds ratio, ref; reference, (-) denotes the variable was not selected by elastic net regularization, for example, Hispanic vs Non-Hispanic White. This implies both Hispanic and Non-Hispanic White can be considered as a group.

* Other includes American Indian/Alaska Native, Asian, Native Hawaiian or Other Pacific Islander

© 2021 Schmidt AL et al. *JAMA Network Open*.

eTable 3. Descriptive Statistics in the ADT Cohort

**Characteristics**	**ADT cohort (*N* = 266)**
30-day all-cause mortality	46 (17)
**COVID-19 severity: Ordinal Scale**	
0 - uncomplicated	100 (38)
1 - hospitalized	85 (32)
2 - ICU	8 (3)
3 - mechanical ventilation	15 (6)
4 - death within 30 days	46 (17)
Unknown/missing	12 (4)
Age median, years (IQR)	74 (65-80) [a]
**Body Mass Index (IQR)** [71 missing]	27.9 (24.9-31.4) [a]
**Race**	
Hispanic	55 (21)
Non-Hispanic Black	35 (13)
Non-Hispanic White	130 (49)
Other*	23 (9)
Missing	23 (9)
**ECOG Performance Status**	
0	81 (30)
1	67 (25)
≥2	46 (17)
Unknown	54 (20)
Missing	18 (7)
**Smoking History**	
Never	112 (42)
Current or Former	122 (46)
Missing	32 (12)
**Cardiovascular Comorbidity**	
No	140 (53)
Yes	108 (41)
Missing	18 (7)
**Pulmonary Comorbidity**	
No	212 (80)
Yes	36 (14)
Missing	18 (7)
**Renal Comorbidity**	
No	203 (76)
Yes	45 (17)
Missing	18 (7)
**Diabetes**	
No	187 (70)
Yes	61 (23)
Missing	18 (7)

© 2021 Schmidt AL et al. *JAMA Network Open*.

**Cancer Status**	
Remission/NED	21 (8)
Active, progressing	57 (21)
Active, responding	71 (27)
Active, stable	88 (33)
Unknown	25 (9)
Missing	4 (2)
**Metastatic Disease present**	
No	80 (30)
Yes	149 (56)
Missing	37 (14)
**Baseline corticosteroid use >10mg oral prednisolone / day**	
No	183 (69)
Yes	54 (20)
Missing	29 (11)
**COVID-19 therapies administered**	
**Remdesivir**	
No	217 (82)
Yes	20 (8)
Missing	29 (11)
**Hydroxychloroquine**	
No	198 (74)
Yes	39 (15)
Missing	29 (11)
**Azithromycin**	
No	196 (74)
Yes	41 (15)
Missing	29 (11)
**Metastatic Disease present**	
No	80 (30)
Yes	149 (56)
Missing	37 (14)
**Prostate Cancer therapies in addition to ADT**	
**1st Generation Androgen Receptor Inhibitor (ARI-1)**	
No	155 (58)
Yes	44 (17)
Missing	67 (25)
**2nd Generation Androgen Receptor Inhibitor (ARI-2)**	
No	162 (61)
Yes	37 (14)
Missing	67 (25)
**Either 1st or 2nd Generation Androgen Receptor Inhibitor (ARI-1/2)**	
No	120 (45)
Yes	79 (30)
Missing	67 (25)
**Abiraterone**	

© 2021 Schmidt AL et al. *JAMA Network Open*.

No	157 (59)
Yes	42 (16)
Missing	67 (25)
**Chemotherapy**	
No	174 (65)
Yes	25 (9)
Missing	67 (25)

NED; No evaluable disease, ADT; Androgen deprivation therapy, ARI-1; 1st Generation Androgen receptor inhibitor; bicalutamide, nilutamide, flutamide, ARI-2; 2nd Generation Androgen receptor inhibitor; apalutamide, darolutamide, enzalutamide, abiraterone.

*Other includes American Indian/Alaska Native, Asian, Native Hawaiian or Other Pacific Islander

© 2021 Schmidt AL et al. *JAMA Network Open*.

eTable 4. Results of Regression Analysis for 30-Day Mortality Between ADT + ARI Compared to ADT, Adjusting for the Variables Selected by the Elastic-Net Regularization With a Mixing Parameter of 1 (LASSO)

**Characteristics**	**Multivariable AOR (95% CI) With PSM**	**Multivariable AOR (95% CI) Without PSM**
**On ARI (ref = No)**		
Yes	0.64 (0.26-1.58)	0.80 (0.38-1.68)
**Age (per 10 years increase)**	**2.68 (1.56-4.60)**	**2.30 (1.44-3.66)**
**ECOG Performance Status (ref = 0/1/Unknown)** [a]		
≥2	**4.63 (1.83-11.75)**	**4.72 (1.86-11.97)**
**COVID-19 treatment, Azithromycin (ref = No)**		
Yes	**-**	**2.69 (1.08-6.70)**

AOR; Adjusted odds ratio, ARI; androgen receptor inhibitor, PSM: propensity score matching, ref; reference, [a] In ECOG Performance Status, 1 vs 0 and Unknown vs 0 were not selected by elastic net regularization; thus, 0/1/Unknown were considered as a group.

© 2021 Schmidt AL et al. *JAMA Network Open*.

eTable 5. Results of Regression Analysis for 30-Day Mortality Between ADT + Abiraterone Compared to ADT, Adjusting for the Variables Selected by the Elastic-Net Regularization With a Mixing Parameter of 1 (LASSO)

**Characteristics**	**Multivariable AOR (95% CI) With PSM**	**Multivariable AOR (95% CI) Without PSM**
**On Abiraterone (ref = No)**		
Yes	0.89 (0.21-3.82)	0.79 (0.28-2.28)
**Age (per 10 years increase)**	**4.61 (1.72-12.38)**	**2.27 (1.43-3.58)**
**ECOG Performance Status (ref = 0/1/Unknown)** [a]		
≥2	**-**	**4.23 (1.78-10.09)**
**COVID-19 treatment, Azithromycin (ref = No)**		
Yes	**4.91 (1.12-21.58)**	**-**

AOR; Adjusted odds ratio, PSM: propensity score matching, ref; reference, [a] In ECOG Performance Status, 1 vs 0 and Unknown vs 0 were not selected by elastic net regularization; thus, 0/1/Unknown were considered as a group.

© 2021 Schmidt AL et al. *JAMA Network Open*.

eTable 6. Results of Regression Analysis for 30-Day Mortality Between ADT + Chemotherapy Compared to ADT, Adjusting for the Variables Selected by the Elastic-Net Regularization With a Mixing Parameter of 1 (LASSO)

Characteristics	Multivariable AOR (95% CI) With PSM	Multivariable AOR (95% CI) Without PSM
**On Chemotherapy (ref = No)**		
Yes	3.37 (0.73-15.55)	3.15 (1.00-9.93)
**Age (per 10 years increase)**	1.49 (0.59-3.79)	**2.56 (1.55-4.21)**
**ECOG Performance Status (ref = 0/1/Unknown)** [a]		
$\geq$2	**7.53 (1.38-41.21)**	**4.09 (1.62-10.33)**
**COVID-19 treatment, Azithromycin (ref = No)**		
Yes	**-**	**2.75 (1.09-6.93)**

AOR; Adjusted odds ratio, PSM: propensity score matching, [a] ref; reference, In ECOG Performance Status, 1 vs 0 and Unknown vs 0 were not selected by elastic net regularization; thus, 0/1/Unknown were considered as a group.

© 2021 Schmidt AL et al. *JAMA Network Open*.

eFigure 1. Patient Selection

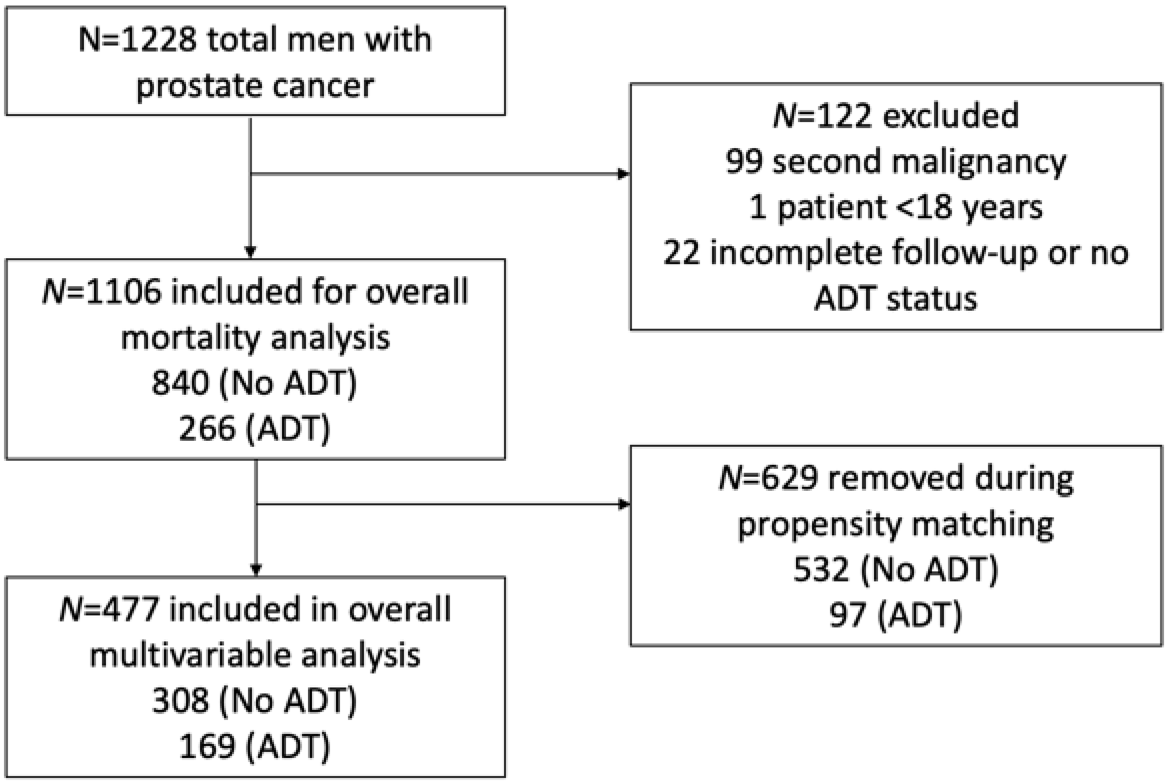

© 2021 Schmidt AL et al. *JAMA Network Open*.

eFigure 2. Loss of Dead30 Events (All Cause 30-Day Mortality) and Standardized Mean Difference of Propensity Scores Between the 2 ADT Groups (on ADT and Not on ADT)

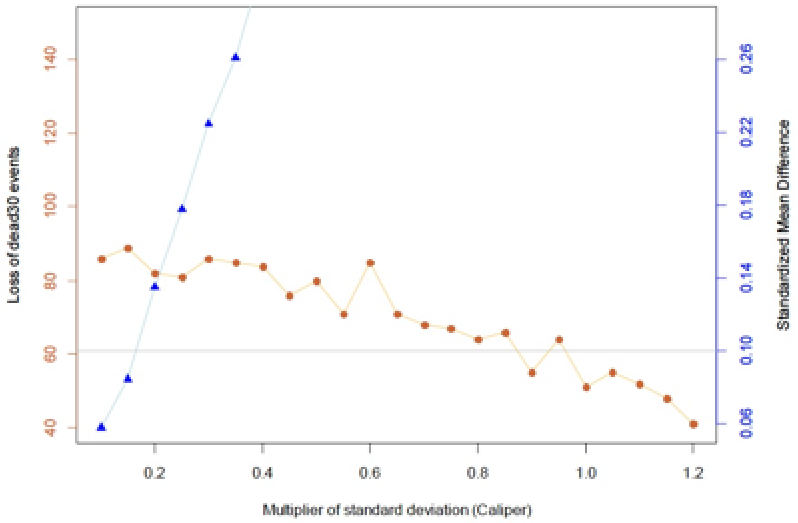

Loss of dead30 events (all cause 30-day mortality) and standardized mean difference of propensity scores between the two ADT groups, which are obtained from two-group propensity score matching using the nearest-neighbor method with a 1:3 ratio (on ADT:not on ADT) and different multipliers of standard deviation.

© 2021 Schmidt AL et al. *JAMA Network Open*.

eFigure 3. Distributions of Propensity Scores of Patients on ADT and Not on ADT Before and After Matching

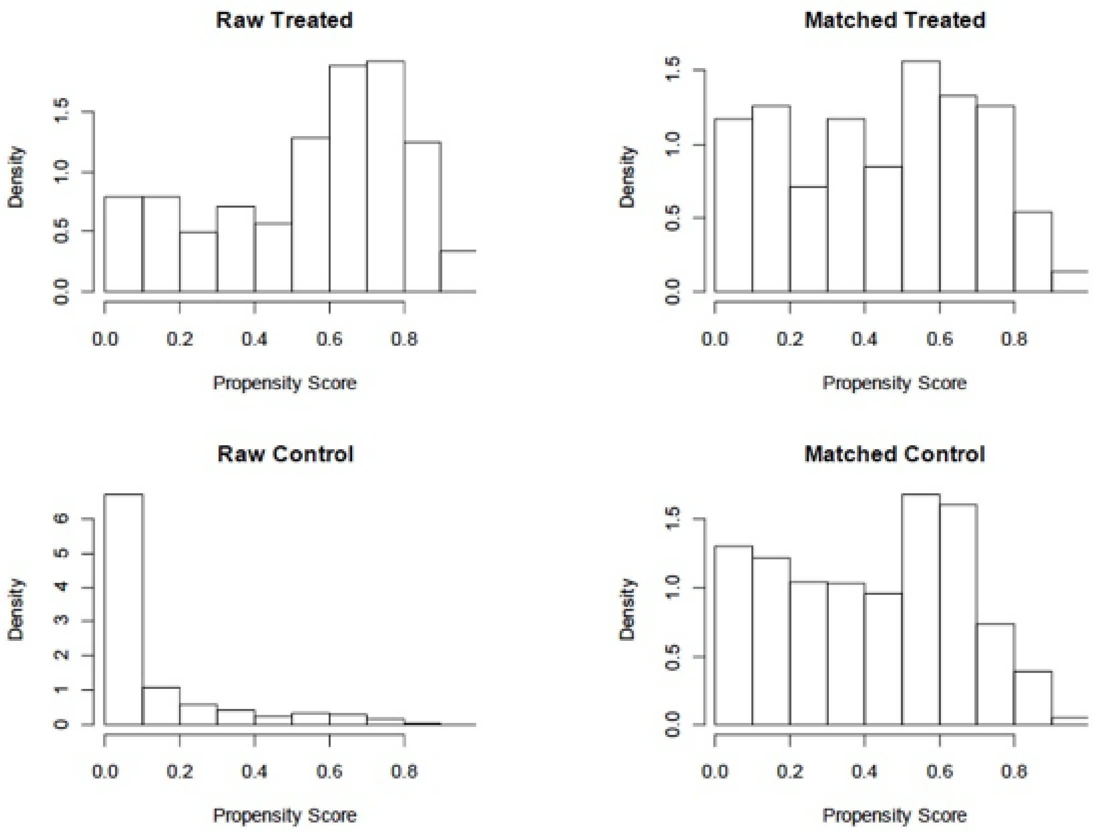

Distributions of propensity scores of patients on ADT and not on ADT before (left) and after (right) matching using the nearest-neighbor method with a 1:3 ratio (patients on ADT and not on ADT) and 0.15 standard deviation.

© 2021 Schmidt AL et al. *JAMA Network Open*.
